# Barriers to access of maternal health commodities among pregnant women in public health facilities in Ubungo Municipal Council, Tanzania

**DOI:** 10.1080/20523211.2023.2300457

**Published:** 2024-01-09

**Authors:** Doris Mollel, Godeliver A. Kagashe, Domina Asingizwe, Stany Banzimana, Shital M. Maru, François Niragire

**Affiliations:** a EAC Regional Centre of Excellence for Vaccines, Immunization, and Health Supply Chain Management, College of Medicine and Health Sciences, University of Rwanda, Kigali, Rwanda; b Medical Stores Department, Dodoma, Tanzania; c School of Pharmacy, Muhimbili University of Health and Allied Sciences, Dar es Salaam, Tanzania; d Department of Pharmaceutical Chemistry, Pharmaceutics and Pharmacognosy, Faculty of Health Sciences, University of Nairobi, Nairobi, Kenya; e Department of Applied Statistics, College of Business and Economics, University of Rwanda, Kigali, Rwanda

## Abstract

**Background:**

Access to maternal health commodities improves maternal outcome and reduce maternal deaths. Tanzania has put in place the fee exemption policy for mothers to access maternal health commodities for free, however, the implementation of user fee exemption has been challenging. Therefore, this study explored the barriers to access of maternal health commodities among pregnant women in public health facilities at Ubungo Municipal Council, Tanzania.

**Methods:**

This was a descriptive qualitative study conducted from January to February 2023. Both focus group discussions and key informant interviews were conducted. These involved 72 pregnant women and 40 health care providers respectively. A purposive sampling technique was used to selected facilities and study participants. Thematic analysis was used to analysis data.

**Results:**

Findings from this study indicated that fear of stigma and discrimination, decision-making autonomy, additional costs and stock out of health commodities were the main barriers to accessing maternal health commodities. Furthermore, both pregnant women and health care providers reported that shortage of health commodities and the inadequacy of service providers, which led to long waiting times, also hinder access to health commodities.

**Conclusion:**

Improving health commodities availability, and increasing the number of service providers are important factors to consider to improve access to maternal health commodities. In addition, factors such as fear of stigma and discrimination should also be addressed to improve maternal health.

## Background

Access to health commodities is a fundamental component in the provision of quality maternal care (Agarwal et al., 2020; Atiga et al., 2023). World Health Organization (WHO) suggests that women have access to prenatal care, delivery services, and emergency obstetric care (EmOC) for improving maternal health and prevent maternal deaths as it is a key foundation for global sustainable development targets (WHO, 2004). Health commodities include medicines, vaccines, medical supplies, medical equipment’s and laboratory/diagnostic consumables as well as other things that may be required for the provision of health services. For maternal care, health commodities involve all equipment, drugs, and other supplies for management of obstetric complications and commodities for prevention, detection, and treatment of hypertension, eclampsia, anaemia, malaria, sexually transmitted diseases such as HIV infection and hepatitis.

Despite various efforts and interventions set by many countries to reduce maternal death, it has been estimated that about one woman of reproductive age dies every two minutes somewhere in the globe, from complications associated with pregnancy and childbirth. The problem is more pronounced in developing countries than in the developed world where it is estimated that over 95 per cent of maternal deaths occur in low and middle-income countries, with 87 per cent occurring in South Asia and Sub-Saharan Africa (Girum & Wasie, 2017). Poor maternal health delivery results in more than half a million maternal deaths during pregnancy, childbirth, or within a few weeks of delivery and some are due to low accessibility of maternal healthcare (Nuamah et al., 2019).

In response to maternal health-related challenges, many governments in developing countries, including many African countries, have adopted user-fees exemption policies on maternal healthcare services to reduce maternal mortality or mitigate the negative impact of user-fees (Hatt et al., 2013; Meessen et al., 2011; Richard et al., 2013). A user fee is a charge imposed by the government for the primary purpose of covering the cost of providing a service, directly generating revenue from the people who benefit from the particular public good or service being provided. The introduction of user fees in public sector organisations in developing nations was driven by the need to raise finances for providing health care services (Prinja et al., 2012). While this is the case however currently, user fees have typically fallen out of favour in Africa as they have been linked to decreased access to healthcare (Robert & Ridde, 2013; Watson et al., 2016).

In public health context, user-fee exemption is internationally recognised as a vital tool for reducing maternal mortality caused by the inability of low-income families to pay for maternal health services to expecting mothers (Hatt et al., 2013; Meessen et al., 2011). In most Sub-Saharan countries, through exemptions, all maternity services that might consume a significant amount of money are automatically granted exemptions (Mubyazi, 2004). To be successful in this, African nations are trying to increase the scope of health insurance coverage by effectively implementing user-fee exemption systems for pregnant women to access maternal healthcare services (Maluka, 2013).

However, It has been indicated that developing countries still face challenges in providing pregnant women who seek out maternal healthcare services with fair access to health care (Macha et al., 2012). Economic factors, poor infrastructures and poor quality of care has been indicated to be barriers faced by most of these countries (Dahab & Sakellariou, 2020). One of the obstacles to the waiver and exemptions’ implementation is the high number of pregnant mothers at healthcare facilities (Ansu-Mensah et al., 2021). This situation places a considerable burden on the health facilities as it increases their budgets due to reimbursement delays from the government to health facilities. Additionally, it has been stated that health facilities often run out stock of health commodities as a result of a high number of exemptions (Maluka, 2013). It has also been reported that some women are sometimes physically and verbally mistreated in addition to being neglected when it comes to receiving maternal health treatments (Kabia et al., 2019). This is because of their low economic status causing health care providers to discriminate them.

In Tanzania, the Ministry of Health started implementing waivers and exemptions in 1994, where the focus, among others, was to ensure that all pregnant women experience equal access to maternal health services (Maluka, 2013). However, studies have shown that waivers and exemptions have brought many challenges in health facilities and limited access to health care services among pregnant women (Kabia et al., 2019; Macha et al., 2012). In addition, although the term ‘policy’ is frequently used, there is no formal guidance on waivers and exemptions, which contributes to the lack of legal power necessary for effective running.

According to Tanzania District Heath Information Software 2 (T-DHIS 2) between 2017 and 2021, the percentage of pregnant women receiving adequate Iron and Folate tablets in the Dar es Salaam region was reported to range from 61.5–100%. The highest rate (100%) was reported in Kigamboni Municipal Council, Dar es Salam region, while the lowest percentage, 61.5%, of pregnant women receiving adequate quantity of Iron and Folate tablets until the next Ante Natal Care (ANC) visit in Dar es Salaam Region occurred in Ubungo Municipal Council. Data from T-DHIS 2 also indicates that the percentage of pregnant women who received Long Lasting Insecticides Nets (LLIN) was 69% (lowest) in the Ubungo Municipal Council and 84% (highest) in the Kigamboni Municipal Council. However, limited information is available on the barriers for access to health commodities among pregnant women in this region. Therefore, this study aimed to explore barriers to accessing health commodities among pregnant women in public health facilities in Ubungo Municipal Council, Tanzania.

## Methods

### Study setting

This study was conducted in the public health facilities offering maternal health services for pregnant women in Ubungo Municipal Council, Dar es Salaam region in Tanzania. The council was selected for this study because the Tanzania Health Information Management System (T-DHIS2), shows that it reported the lowest percentage of pregnant women receiving adequate quantities of Iron and Folate tablets until the next ANC visit and the lowest rate of pregnant women who received Long Lasting Insecticides Nets (LLIN) between 2017 and 2021.

### Study design

The was a descriptive, qualitative study conducted from January to February 2023. Focus Group Discussions (FGD) and In-Depth Interviews (IDI) were employed to collect data from pregnant women and healthcare providers on the barriers to accessing health commodities among pregnant women in public health facilities. This enabled direct exploration, analysis, and description of the barriers to accessing health commodities among pregnant women in Ubungo Municipal Council, Tanzania.

### Study population and sampling

The population for this study were pregnant women attending public maternal health care clinics and health care providers providing maternal health care among pregnant women at public health facilities. These facilities were obtained from a list of public health facilities that provide maternal healthcare services obtained from the Ministry of Health (MoH), Tanzania Mainland and the President’s Office – Regional Administrative and Local Government (PO – RALG). Purposive sampling was used to obtain ten (10) health facilities with the highest volume of pregnant women attending maternal healthcare clinics. These facilities included Kimara, Sinza, Mbezi, Makurumla Health Centers, and Goba, Manzese, Makuburi, Malamba Mawili, Amani, and Mavurunza dispensaries.

A convenient sampling technique was used to recruit pregnant women coming for maternal healthcare services at the selected health facilities. This study sample included pregnant women who were easily accessible at healthcare facilities and accepted to participate. They were asked on important questions including the number of visits they have attended on particular facility and demographic questions in order to determine their eligibility for the discussion. The service providers helped the researcher to inform the pregnant mothers about the study and those who wished to participate voluntarily were recruited for the interview in a separate room in the facility.

For the In depth Interviews, forty (40) healthcare providers (four from each selected 10 facilities), providing maternal healthcare to pregnant women were purposively selected based on their experience pertaining to the research problem. They included health facility in-charges who are also clinicians, facility pharmacists, dispensers, and RCH nurses and midwives.

### Data collection tools and procedure

A focus group guide and interview guide were developed based on the research objective. Before starting the interviews, the visited health facilities were informed about conducting field observation to allow voluntary participation and informed consents. For the FGDs, the visited health facility authorities helped the researcher to meet with pregnant women coming for maternal healthcare services at the selected health facilities to request their consent to join a focus group discussion. A total of 10 FGDs were conducted from selected facilities involving 72 pregnant women in total and ranging from 6 to 11 each FGD.

In addition, a total of 40 In-depth interviews were conducted with 40 healthcare providers from all the selected facilities. The participants for IDIs were health facility in-charges, facility pharmacists, dispensers, and nurses and/or midwives who works in Reproductive and Chid Heath Unit (RCH).16 Health Care Providers were males where 24 were females. Their ages ranged from 25 to 54 years with work experience between 4–32 years. IDIs were conducted in a private space to allow participants to be comfortable in sharing information. FGDs and IDIs were collected using a tape recorder with permission from the participants, and notes were taken as appropriate. Kiswahili language was used for both FGDs and IDIs to avoid any language barrier.

### Protocol and ethical consideration

The researcher obtained approval from the University of Rwanda. After obtaining permit, the researcher sought permission to conduct the study form Presidents Office-Regional Administrative and Local Government Ministry, Regional Administrative Secretary, Municipal Director’s Office and Facility levels. To make sure the informants agreed to engage voluntarily in the study, the researcher met with them before the interview and obtained their informed consent. Information was kept completely confidential and only shared with appropriate parties by preventing unauthorised individuals from acquiring study data. The identity of the anonymous informants were kept strictly confidential because no names were taken down throughout the interviews and discussions.. The participants in the study received a thorough explanation of the study's details. The study's methodology, advantages, risks, and voluntary participation were all covered in detail. The researcher maintained an honest and open demeanour throughout the whole data collection process. This study did no harm because ethical issues were taken into account.

## Data analysis

Data was analysed manually using the thematic analysis method, which systematically identifies, organises, and offers insight into patterns of meaning (themes) across a dataset (Reise & Moore, 2012). By focusing on meaning across a dataset, the thematic analysis allowed the researcher to see and make sense of collective or shared meanings and experiences (Reise & Moore, 2012).

In this study, all the interviews and FGDs were first transcribed and translated from Kiswahili to English. An inductive and a deductive analysis were performed. The inductive method is a bottom-up strategy that uses the raw data collected to generate codes. The approach enabled for the emergence of new theories, concepts, and ideas because the study was explanatory in nature. The analysis involved re-reading the translated data to be familiar with the data and then allocating preliminary codes to generate the themes from the codes by joining similar codes. Each section that related to the critical aspects was given a tentative code. The tentative codes were progressively improved and revised to create the final list of codes. The main themes and codes were used to describe the findings along with the study's objectives. The first author generated the initial list of codes. The second author reviewed and revised the list as appropriate. The whole team then reviewed, revised, and agreed on the final list of codes, as well as the categories, and themes.

## Results

### Demographic characteristics of the study participants

Table 1 *indicates* demographic data for In depth Interview Participants (Health care providers): 24 participants (60%) were female, 22 participants representing 50% had diploma level education, 28 were married (70%) and 35% had a work experience of 11–15 years.

**Table 1.** Demographic data for In-depth interviews participants (health care providers).

Sex	Frequency	Percent (%)
Male	16	40
Female	24	60
Age	Frequency	Percent (%)
25–35 years	11	28
36–45 years	21	53
46 years and above	7	18
Level of Education	Frequency	Percent (%)
Certificate	10	25
Diploma	22	55
Degree	8	20
Marital Stasus	Frequency	Percent (%)
Single	12	30
Married	28	70
Divorced	2	5
Work Experience	Frequency	Percent (%)
0–5 years	2	5
6–10 years	13	33
11–15 years	14	35
16–20 years	7	18
above 20 years	4	10

Among the pregnant women, 26 were below 25 years, and 46 were above 25 years. Thirty-three women had primary education, 36 had secondary education, and 3 had degree-level education, Table 2 indicates the details.

**Table 2.** Demographic data for FGD participants (pregnant women).

Age	Frequency	Percent (%)
below 25 years	26	36
above 25 years	46	64
Level of Education	Frequency	Percent (%)
Primary	33	46
Secondary	36	50
University level	3	4
Marital Stasus	Frequency	Percent (%)
Single	24	33
Married	46	64
Divorced	2	3

Eight themes emerged from the study and are presented as the main headings in this result section.

### Poor health literacy about maternal health

This study found that lack of awareness about maternal health impedes maternal health commodity-seeking behaviour. It is revealed that poor maternal knowledge about pregnancy and health services offered during pregnancy is a barrier to accessing maternal health commodities among pregnant women as it leads to an inadequate understanding of the effectiveness of maternal health services and related items.

Some respondents expressed that pregnant women do not utilise exempted maternal health commodities because they do not understand the benefits. Pregnant women with maternal knowledge are more likely to access maternal health commodities than their counterparts, pregnant women without maternal knowledge. One health facility in charge expressed:

I think health illiteracy about pregnancy is an issue. This is why we try hard to educate them so that they understand and have proper information about pregnancy. Some pregnant women ignore using iron tablets because they do not understand the importance of taking iron during pregnancy and the consequences of not taking these medicines (IDI with Health Facility in charge).

### Economic status of pregnant women

Even though maternal health services are not charged during pregnancy, it was reported that additional costs incurred to obtain them affect the accessibility of health commodities among pregnant women in public health facilities. Most participating pregnant women and service providers explained that some clients miss or postpone their usual ANC appointment because transport costs constrain them and sometimes cover some tests or services that are not available in health facilities. During the interviews, one participant said:

Economic status influences access because we have different financial status. So, you sometimes skip medication for the entire month if you don’t have money for transport to come to the clinic (FGD 2).

In addition, the study results show that some pregnant women use the money they have to buy household needs like food rather than spending it on other services associated with maternal health commodities accessibility such as transport. They have to choose to prioritise basic needs for their households rather than maternal health commodities.

Life in urban areas is tough. Some women can’t even afford to get a meal. Therefore, some decide not to come because they don’t have money for transport to go to the facility (IDI/ facility in-charge /aged 40).

### Fear of stigma and discrimination among HIV-positive mothers

This study reveals that the persistent social stigma and discrimination are barriers to exempting maternal health commodities among pregnant women. If a pregnant woman is tested and found HIV positive, she becomes afraid to disclose her HIV status to the spouse to continue attending ANC and CTC with the fear that their spouses will run away when they know their wives are living with HIV. Thus, some of these pregnant women decide to hide their pregnancies and stop going for exempted maternal commodities and HIV testing. During in-depth interviews, participating service providers said:

Some pregnant women are afraid to allow US to disclose their HIV status to their spouse when tested and found HIV positive. They are afraid that their spouses will run them away if we tell them. And yes, some of them are also run away by their spouses and left with the burden of the families if their HIV status is disclosed (IDI with RCH nurse)

In addition, findings indicated that stigma and discrimination from society also hinder access as it leads to poor adherence to medications, particularly ARVs. One participant said:

Some pregnant women living with HIV stop coming to take the ARVs, fearing that people will despise and discriminate against them if they come to know that she is living with HIV (IDI with Laboratory Assistant)

### Decision-making autonomy

From this study, it was revealed that decision-making autonomy influences maternal health commodities accessibility among pregnant women. Pregnant women with decision-making readiness about their health status can easily access maternal health commodities that spouses have to make decisions for them. During the interviews, some participating service providers indicated that some pregnant women are not permitted by their spouses to come to the facility for ANC. This means that some husbands must decide for their wives to go to the facilities for maternal health services utilisation. During the interviews, participants said:

The biggest challenge is the lack of decision-making power among pregnant women. Some pregnant women will tell you that they have failed to come to the health facility because their spouses were not around or they did not get permission from their spouses to go for the services (IDI with facility in charge).

Similar results emerged out of the focus group discussions. Some of the pregnant participants revealed that they occasionally miss their clinical appointments because their husbands prevent them from visiting the facilities. During the discussion, one of the participating pregnant women explained:

Most of us women do not have the power to make decisions about our health matters. For example, some women miss their clinical appointments because their husbands are not available, and they are restricted from going out of their homes (FGD 4).

### Stock out of maternal health commodities

It was reported that sometimes there is a stockout of exempted maternal health commodities at the health facilities, and this hinders their accessibility. Some pregnant women stop coming to attend their usual ANC visits when they notice that there is a stockout of maternal health commodities for prevention, detection, and treatment of hypertension, eclampsia, anaemia, malaria, and sexually transmitted diseases such as HIV infection and hepatitis for pregnant women. Both participating pregnant women and service providers explained that some pregnant women tend to give up attending their usual ANC visits when they are returned or told to buy the medicines from private pharmacies during frequent maternal medication stockouts. During the interviews and discussions, some participants said:

The challenge is a frequent shortage of exempted maternal health commodities such as Sulfadoxine/Pyrimethamine tablets, Ferrous and Folic acid tablets and mosquito nets with long-lasting insecticide treatment and Haemoglobin tests. This means that most pregnant women do not use mosquito nets because they are unavailable here. Some pregnant women do not come to public facilities because they face a shortage when they arrive (IDI with RCH nurse)

Further, the inadequate dosage of maternal health commodities due to running out of stock was also found to be a barrier to access to health commodities in public health facilities, as explained by one participant:

Some pregnant women are given half of the needed dosage when they come to this facility because of a shortage. For example, instead of being given iron tablets for a month, they are given half of it **(**IDI with a Facility Pharmacist)

In addition, participants indicated that some pregnant women tend to give up attending their usual ANC visits when they are told to do some tests in other health facilities because of maternal health commodities stockout. During the FGD, one participant said:

Some equipment is not available here. I remember the day I came to start ANC, I was forced to go to the private facility because there was no ultrasound there. I didn’t do it because I could not afford it from the private facility (FGD 7).

Furthermore, it was revealed that some high-volume health facilities experience early stockout because more pregnant women than maternal health commodities are available at the facilities.

Pregnant women are free to attend ANC services at any public health facility, but every health facility has its budget for the projected population that should serve. Hence, some health facilities serve pregnant women not within their catchment area. In the end, this leads to early shortage therefore, some pregnant women miss some maternal health commodities. During an interview, one of the participating service providers was heard saying:

There is much politics in this. Pregnant women are told to get services anywhere they want. This is not practical because every facility has its projections and budget. They are receiving clients from other areas whom you did not budget for leading to a shortage, and some women miss commodities **(**IDI with RCH nurse).

### Limited space to accommodate ANC services

The findings revealed that some ANC rooms are overcrowded and do have not enough waiting areas for pregnant women. Some pregnant women become frustrated and give up their maternal health clinics because there is nowhere to sit comfortably waiting for the services. The study results further showed that some pregnant women are even forced to stand in the sun or sit on the paving because the waiting area is overcrowded and there are no chairs; hence, some opt-out from waiting and leave without receiving maternal health commodities. During interviews and FGDs, some participating pregnant women and service providers from some of the visited healthcare facilities said:

There is a shortage of waiting areas at this facility. You are forced to stand in the sun because the whole place is occupied. Some women leave without being provided with the service because they get tired (FGD 1).

### Availability of service providers

The findings revealed that a shortage of health personnel is a barrier to maternal health commodities among pregnant women since it increases the facility's long waiting times. Therefore, some pregnant women gets disappointed and decide not to attend their scheduled clinic visits as a result of such experience. One of the participating pregnant women had this to say during the focus group discussion:

The biggest challenge is that there aren’t adequate service providers at this facility. We spend much time at the facility because there are few service providers. Some women discontinue their maternal health services because they do not want to spend much time at the facility (FGD 6).

Shortage of staff resulted in high client load to service providers where service providers get tired and are forced to skip some services. This means that some pregnant women get insufficient maternal commodities because there are insufficient service providers to offer sufficient services and items. A participating pregnant woman explained:

I think there are not enough service providers. You find that a service provider serves for a long time, and the queue does not even end. S/he ends up very tired and skips some tests. For example, s/he will ask you if the baby is kicking. If you say yes, s/he does not examine the baby’s heartbeat (FGD 5).

### Exemption mechanisms for maternal health commodities

Some health facilities fail to provide exempted maternal health commodities because the exemption mechanism is not implementable. Some participants noted that some maternal health commodities are to be provided free to pregnant women while they are to be paid for by health facilities from the Medical Stores Department. During interviews, a participant said:

The health facilities are overloaded. Not every maternal health commodities is provided free from the MSD as vertical items. We are blamed by pregnant women when they miss commodities, but they do not know if we pay to get those commodities (IDI with a facility pharmacist).

## Discussion

This study explored the barriers to accessing maternal health commodities among pregnant women in public health facilities at Ubungo Municipal Council, Tanzania. The current study reported different obstacles, and these are related to either the pregnant woman (including poor health literacy, economic status, fear and stigma of discrimination for HIV positive mothers, as well as decision-making power) or health facility/system (including stockout of commodities, limited space, insufficient health providers, and as well as exemption mechanisms).

The current study indicates that lack of knowledge about the importance of maternal health commodities, as well as consequences and complications associated with not taking these commodities, hinders access to maternal health services. Also, economic status, decision-making autonomy, and fear of stigma and discrimination of pregnant mothers hinder access to maternal health services. Similarly, studies conducted in Tanzania identified financial constraints such as transportation costs and costs of maternity services as major hindrances to maternity health-seeking behaviour during the first trimester and after delivery (Kristoamani & Mahiti, 2020; Panga & Mosha, 2022). The study done in Nigeria in 2021 is also in line with the findings from this study which indicated that the economic status of pregnant women, decision-making autonomy, and fear of stigma and discrimination influence the uptake of maternal health commodities by pregnant women (Macha et al., 2012). Moreover, these results agree with a study conducted in Madagascar in 2021 which revealed that financial issues related to the costs of health facility commodities influence the likelihood of accessing ANC commodities among pregnant women (Dahab & Sakellariou, 2020). Similarly, a study conducted in Geita, Tanzania in 2020 on community perceptions of the place of childbirth found that gender-based roles and economic factors influence pregnant women’s decisions on healthcare utilisation (Ansu-Mensah et al., 2021). District Medical Offices and other health stakeholders should emphasise promoting maternal health literacy and reducing stigma and discrimination through social mobilisation campaigns to raise societal awareness of maternal healthcare.

Stock out of maternal health commodities is another barrier to accessing maternal health commodities among pregnant women in public health facilities reported in this study. This means that some pregnant women will stop coming to attend their usual ANC visits when they notice that they are not provided with the needed health commodities at the facility. These results are consistent with a study conducted in 2015 in rural Tanzania that indicated the unavailability of maternal services, in particular health commodities, is a barrier to accessing health care services (Kabia et al., 2019). Additionally, a study conducted in Madagascar revealed that women's utilisation of maternal healthcare services at the health facilities was influenced by the availability of health commodities (Dahab & Sakellariou, 2020). These findings are concurred by various studies conducted in Tanzania between 2018 and 2020 which revealed that the availability of medical supplies and drugs influences pregnant women to utilise existing maternal health services (Ansu-Mensah et al., 2021; Neke et al., 2018).

Stock outs are attributed by high number of pregnant women attending ANC services in relation to the budget allocated, stock out at the Medical stores Department and inadequate utilisation of health commodity funds. To address this, Health Facility Management Teams should ensure that there is timely ordering of health commodities to avoid stock outs and that funds allocated for health commodities are utilised as planned. The Government should also capacitate Medical Stores Department to ensure that maternal health commodities are available all the time.

Limited waiting space was another barrier that was revealed to hinder access to maternal health commodities in public health facilities. From the study results, it can be discussed that some pregnant women can give up their maternal health clinics because there is nowhere to sit comfortably waiting for maternal health commodities. These results are similar to studies done in Tanzania in 2020 which pointed out that the availability of maternity waiting areas at the health facility influenced accessibility of maternal health services among pregnant women (Kristoamani & Mahiti, 2020; van Pelt et al., 2020). Therefore, improving health facilities infrastructure by providing a comfortable environment for patients during service delivery could improve access to maternal health commodities.

The current findings reported a shortage of service providers increases queues at the clinic waiting area and waiting times which in turn frustrates pregnant women. In the end, some pregnant women may leave the facility without the intended service and/or opt to stop attending their maternal health clinics as required because they do not have time to wait long at the clinics. These results are in line with the study conducted in Tanzania in 2022 that revealed that shortage of health care providers is a major reason for long waiting time at the health care facilities hence barrier to access of maternal health services (Panga & Mosha, 2022). Similarly a study by conducted by Mukumbang et al in 2017 stated that availability of medical staff is among the influencing factors for the access to health services (Mukumbang et al., 2017). The study concludes that having adequate service providers is important as it would reduce queues and improve access to maternal health commodities.

Moreover, it is revealed from this study results that policies and regulations are vital in promoting access to exempted maternal health commodities by pregnant women in public health facilities. This is similar to the framework explained that policies and regulations are vital to shaping the availability, quality, and affordability of maternal health commodities (JSI, 2014).

Policies determine the availability and accessibility of health facilities. The government must ensure there are enough health facilities which are also easily accessible to pregnant women. women. Also, policies determine the availability of health workers and medical supplies. It is the government’s responsibility to ensure there are adequate health workers and maternal health commodities in the health facilities. Through its policies, the government is responsible for employing enough service providers and providing adequate maternal health commodities to its public health facilities.

### Study imitations

This study was carried out inside a set of purposively selected public health facilities and thus, they cannot be generalised to all health facilities in Tanzania. Only pregnant women who attend ANC visits in a public health facility were involved. However, pregnant women who visited private health facilities can provide additional relevant data. In addition, the study was conducted in a health facility setting with a high volume of pregnant women in Ubungo Municipal Council, which is an urban setting. Thus, this study does not address pregnant women who are attending low-volume health facilities or in different sociocultural and geographical diversities.

## Conclusion

This study explored the barriers to accessing maternal health commodities among pregnant women in public health facilities at Ubungo Municipal Council, Tanzania. The current study reported different barriers, and these are either related to the pregnant mother or health facility. Those related to the pregnant mother include poor health literacy, economic status, fear and stigma of discrimination for HIV-positive mothers, as well as decision-making power. In addition, health facility-related factors include stockout of commodities, limited space, insufficient health providers, and as well as exemption mechanisms. The barriers presented in this study serve as basis and may be used by further research to quantity potential association with the accessibility of the health commodities.

## Author contributions

DM designed the study, collected data and drafted the manuscript. GAK participated in the design and implementation of the study. DA, SB, SMM, and FN participated in data analysis, drafting the manuscripts, and critically reviewed and revised the manuscript. All authors read and approved the final manuscript.

## Data Availability

Transcripts used during the study are available from the corresponding author on reasonable request.
